# Signatures of immune reprogramming in anti-CD52 therapy of MS: markers for risk stratification and treatment response

**DOI:** 10.1186/s42466-019-0045-x

**Published:** 2019-12-13

**Authors:** Laura Bierhansl, Tobias Ruck, Steffen Pfeuffer, Catharina C. Gross, Heinz Wiendl, Sven G. Meuth

**Affiliations:** Department of Neurology with Institute of Translational Neurology, University Hospital Muenster, Albert-Schweitzer-Campus 1, 48149 Muenster, Germany

**Keywords:** Relapse-remitting multiple sclerosis, Alemtuzumab, CD52, Biomarker, mechanism of action, secondary autoimmune disease, Disease-modifying therapy, risk stratification

## Abstract

**Background:**

Multiple sclerosis is one of the most prevalent neurological diseases in young adults affecting over 2 million people worldwide. Alemtuzumab is a highly effective therapy in relapsing remitting MS. Alemtuzumab is a monoclonal CD52 antibody that proved its efficacy against an active comparator (interferon [IFN]-β1a) in a phase II trial and two phase III trials regarding clinical and MRI outcomes. Nevertheless, the exact mode of action is still unknown. Alemtuzumab is commonly associated with secondary autoimmune disorders significantly affecting the risk-benefit ratio. Therefore, new biomarkers predicting treatment response and adverse events are urgently needed. This study aims to further elucidate the mechanism of action of the neuroprotective potential of alemtuzumab in relapsing-remitting multiple sclerosis (RRMS).

**Methods/Design:**

This is a 3-year multicentre, explorative study including overall 150 patients comprising three different groups: (i) de novo patients prior and after alemtuzumab treatment initiation, (ii) patients under alemtuzumab treatment and (iii) patients requiring more than two alemtuzumab infusions. Peripheral blood and serum samples will be collected semi-annually for several in vitro/ex vivo assays to detect and characterize immune cells including their functional activity. Furthermore, data of MRI scans and disease-related impairment (using EDSS and MSFC), as well as the number and time of relapses, will be assessed. The clinical study is registered at clinicaltrials.gov (NCT04082260).

**Perspective:**

Our study will provide deep insights into the underlying immunological changes in a longitudinal analysis of alemtuzumab treated RRMS patients. By combining clinical, radiological and functional immune-phenotype data, we will be able to identify biomarkers and/or immune signatures predicting treatment response and adverse events. Thereby, the understanding of the mechanisms of action of alemtuzumab will improve its efficacy and safety for present and future patients.

## Background

Multiple sclerosis affects over 2 million people worldwide and most commonly young adults usually above the second and below the fourth decade of life [1]. Pathological hallmarks of MS are inflammation, demyelination, oligodendrocyte death, axonal damage and neurodegeneration [2]. Therefore, many therapies have been established to modify the adaptive immune system to prevent progression of the disease. In 1991 the first MS patient had been treated with alemtuzumab, a humanized, monoclonal antibody selectively targeting CD52 which is expressed by T- and B-lymphocytes, NK cells, dendritic cells, monocytes and macrophages [3]. Since 2013 alemtuzumab has been approved for the treatment of RRMS by the EMA (European Medicine Agency) under the name Lemtrada®.

The two phase 3 approval trials CARE-MS I (Comparison of Alemtuzumab and Rebif Efficacy in Multiple Sclerosis) and CARE-MS II compared alemtuzumab with IFN-β1a therapy and included treatment- naïve or pre-treated RRMS patients. The 5-year evaluation of efficacy and safety data indicated that the annualized clinical relapse activity was reduced, most patients showed no evidence of disease activity, and the extent of brain volume loss was slowed, even though 68.5% [CARE-I] and 59.8% (CARE-MS II) of patients did not receive an additional alemtuzumab course after their second annual cycle [4, 5].

Alemtuzumab leads to sustained quantitative and qualitative changes within the immune system and is therefore considered a short-pulse immune reconstitution therapy, where 12 mg/day is i.v. administered for 5 consecutive days and followed by a 3-day treatment 12 months later. This treatment results in an initial depletion of lymphocytes followed by a slow repopulation. It was observed that B cells and NK cells recur fast within a matter of 3 to 6 months, while peripheral T cells are mostly undetectable for 12 to 18 months [6].

The most common adverse effects of alemtuzumab are infusion-associated reactions and infections, which occur at the highest rate after the first treatment course. Most relevant are secondary autoimmune events, including thyroid disease, immune thrombocytopenia and nephropathy [7]. The experiences of these trials led to the implementation of an extensive safety measures allowing for early recognition and therapy of adverse events. However, recently in April 2019 the EMA (European Medicine Agency) has initiated a review of alemtuzumab therapy, since new side effects were reported including immune-mediated diseases (autoimmune hepatitis, hemophagocytic lymphohistiocytosis) and cardiovascular disorders (lung bleedings, heart attacks, strokes and cervicocephalic arterial dissections). While the review is ongoing alemtuzumab use was restricted to patients with active relapsing-remitting multiple sclerosis despite a full and adequate course of treatment with at least two other disease-modifying therapies or to patients where all other disease-modifying therapies are contraindicated [8].

Alemtuzumab has demonstrated to be a highly effective therapy with a safety profile requiring specific precautions defined by a risk management program for RRMS. However, the mechanisms of action remain largely unknown [9]. Combining clinical, radiological and immunological data the present multicentre, explorative phase IV study aims to elucidate further insights into the mechanisms of action of alemtuzumab treatment and to identify biomarkers for treatment efficacy and safety. This study is supported by the German Federal Ministry for Education and Research (BMBF) as a project within the German Competence Network Multiple Sclerosis (Kompetenznetzwerk Multiple Sklerose, KKNMS).

## Methods

### Aim of the trial

To elucidate the mechanism of action and neuroprotective potential of alemtuzumab we aim to combine clinical data (MRI, EDSS, MSFC) with in vitro (e.g. isolation/stimulation of immune cell subsets, cytokine production, effector functions) and ex vivo (ten-colour flow cytometry from peripheral blood of alemtuzumab treated patients) data.

### Study description and study design

This study is a multicentre explorative phase IV study to elucidate further insight into the mechanisms of action of alemtuzumab treatment in patients with RRMS. We aim to include around 300 alemtuzumab treated MS patients. The study will be continued for 3 years with regular blood sampling every 6 months. The study will be conducted in multiple KKNMS centers and is supported by the German Federal Ministry for Education and Research (BMBF). Recruitment started in January 2017. Each patient’s study participation will be 3 years. With study inclusion and every 6 months during this time, there will be blood sampling and clinical assessments (anamnestic data, EDSS, MSFC) at the study center. MRI scans are part of the routine diagnostic of MS patients. The data from these scans will be correlated with clinical and experimental parameters. Scheduling will be based on the date treatment is initiated. If a patient is eligible for study participation, the baseline assessments listed in Table 1 are performed before treatment with alemtuzumab is started. If a patient experiences a relapse after clinical baseline assessments (EDSS, MSFC) and prior to initiation of alemtuzumab, the respective examination must be repeated before treatment is started. During the study period the patient will be examined every 6 months following the regular study procedures (see Table 1). All collected samples and data will be organized by professional biobanking which results in a highly controlled and standardized collection, storage and analysis of the biological samples.

**Table 1:** Regular Study Procedures

Baseline assessments Month 0	Month 6, 12, 18, 24, 30 and 36 after treatment initiation
• Demographic information (date of birth, gender)	
• Determination and documentation of eligibility by the Investigator or authorized representative (according to eligibility criteria)	
• Relevant medical history: e.g., lumbar puncture, MRI, medication history and current medications, medical conditions/surgical history, clinical episode history	
• Physical examination and documentation of vital signs	• Physical examination and documentation of vital signs
• Adverse events and concomitant medication	• Adverse events and concomitant medication
• MSFC	• MSFC
• EDSS	• EDSS
• Collection of peripheral blood and serum for several in vitro and ex vivo assessments (8 × 9 ml EDTA blood, 2 × 9 ml serum) as part of the routine blood assessments (including complete blood count, serum creatinine levels, tests of thyroid function)	• Collection of peripheral blood and serum for several in vitro and ex vivo assessments (8 × 9 ml EDTA blood, 2 × 9 ml serum) as part of the routine blood assessments (including complete blood count, serum creatinine levels, tests of thyroid function)

The clinical study is registered at clinicaltrials.gov (NCT04082260). It will be performed in accordance with the approval of the local ethics committee (Ethik-Kommission der Ärztekammer Westfalen-Lippe und der Westfälischen Wilhelms-Universität Münster, 2014–398-f-S), the requirements of the current German drug law (“Arzneimittelgesetz”), the current legal provisions regarding data protection, and the principals of Good Clinical Practice. All participants are obliged to hand in the signed informed consent form (ICF).

### Arms and interventions

The patient population will be patients with active RRMS. Different patient groups will be included: (i) de novo patients (until 04/2019) (ii) patients under alemtuzumab treatment (iii) patients requiring more than two alemtuzumab infusions.

### Outcome measures

#### Primary endpoints

Absolute and relative change of cell-counts compared to baseline of T cell subsets (CD4 and CD8 positive T cells: naïve T cells, T effector cells, T memory cells, regulatory T cells; T-helper subsets: Th1, Th2, Th17), B-cell subsets (recent bone marrow emigrants, mature naive, memory B cells and plasma cells), natural killer cells (CD56^bright^, CD56^dim^), natural killer T cells, antigen-presenting cells (Dendritic cells: CD303^+^ plasmacytoid, CD11c^+^ and CD141^+^ myeloid dendritic cells, monocytes and macrophages) and myeloid-derived suppressor cells in the peripheral blood samples at indicated time points of sampling will be considered as primary endpoints. Furthermore, markers of autoimmunity in the serum (ANA, cANCA, pANCA, anti-dsDNA, anti-TSHR, anti-TPO, RF, anti-CCP, anti-GBM and anti-platelet antibodies) will be considered as further primary endpoints (see Table 2).

**Table 2:** Endpoints of the study

Primary Endpoints	
• T cell subsets: - CD4 and CD8 positive T cells: naïve T cells, T effector cells, T memory cells, regulatory T cells - T-helper subsets: Th1, Th2, Th17	
• B cell subsets: - Recent bone marrow emigrants, mature naïve, memory B cells - Plasma cells	
• Natural killer cells: - CD56bright, CD56dim - Natural killer T cells	
• Antigen-presenting cells: - Dendritic cells: CD303^+^ plasmacytoid, CD11c^+^ and CD141^+^ myeloid dendritic cells - Monocytes and macrophages	
• Myeloid-derived suppressor cells	
• Markers of autoimmunity in the serum (every 6 months): - ANA, cANCA, pANCA, anti-dsDNA, anti-TSHR, anti-TPO, RF, anti-CCP, anti-GBM, anti-platelet antibodies	
Secondary Endpoints	
• Activation status of cell surface receptors assessed by flow cytometry: - Relative and absolute change from baseline of mean fluorescence intensity (MFI) and the proportion of positive cells regarding CD25, HLA-DR, LFA-1, CD29, CD69, CD71 expression	
• Expression of co-inhibitory molecules assessed by flow cytometry: - Relative and absolute change from baseline of MFI and of the proportion of positive cells regarding PD-1 = CD279, ICOS = CD278, TIM-3, CTLA4 expression	
• Effector functions of CD4 and CD8 positive T cells: - Relative and absolute change from baseline of the results of cell proliferation assays assessed as the percentage of proliferated cells - Relative and absolute change from baseline of cytokine production measurement assessed as concentration - Relative and absolute change from baseline of the cytolytic activity assessed by flow cytometry measurement of MFI and proportion of positive cells regarding Granzyme B, Perforin and CD107a expression - Relative and absolute change from baseline of intracellular calcium response assessed as a ratio	
• Migrational capacity: - Relative and absolute change from baseline MFI and the proportion of positive cells assessed by flow cytometry expression analysis of CD11a, CD31, CD44, CD49d, CCR5, CCR6, CCR7 - Absolute and relative change of cell numbers of migrated cells compared to baseline assessed in an in vitro model by flow cytometry analysis	
• High-throughput TCR- and BCR sequencing of the T- and B cell rep-ertoire concerning the expansion of distinct clones: - Qualitative comparison of the distribution of CDR3 sequences	
• Regulatory T-cell function: - Relative and absolute change from baseline in production of TGF-beta and IL-10 of CD4^+^CD25^+^FOXP3^+^ regulatory T cells - Suppression of T cell proliferation: Relative and absolute change from baseline in responder T cell proliferation assessed by suppression assays	

##### Cell isolation

PBMCs (peripheral blood mononuclear cells) will be isolated from peripheral blood by centrifugation on a Lymphoprep™ (Fresenius Kabi Norge AS) density gradient. If needed cells will be aliquoted and cryo-preserved in liquid nitrogen, except for the analysis of immune cell composition where freshly isolated cells will be used.

##### Flow cytometry

The identification and quantification of leukocyte subsets will be done by flow cytometric analysis of PBMCs as described before [10]. The BD Cytofix/Cytoperm™ Fixation/Permeabilization Solution Kit with BD GolgiPlug™ (BD Biosciences) will be used for intracellular stainings according to manufacturer’s protocols. Cells will be analyzed on a Navios Flow Cytometer (Beckman Coulter).

##### Markers of autoimmunity

In order to better assess and monitor the potential for autoimmune disease, the serum will be tested for the following markers of autoimmunity: ANA, cANCA, pANCA, anti-dsDNA, anti-TSHR, anti-TPO, RF, anti-CCP, anti-GBM and anti-platelet antibodies. Samples for autoimmunity testing will be obtained before the start of treatment with alemtuzumab and every 6 months thereafter. There will be additional testing in case of other suspected autoimmune diseases.

#### Secondary endpoints

Secondary endpoints will further characterize the T- and B-cell population in the peripheral blood (every 6 months) (see Table 2). Therefore, the activation status of cell surface receptors, as well as expression of co-inhibitory molecules, will be analyzed by flow cytometry. Furthermore, effector functions of CD4 and CD8 positive T cells will be assessed (e.g. cell proliferation, cytokine production, cytolytic activity and intracellular calcium response). Using flow cytometry expression analysis and in vitro models for migration, the migrational capacity will be analyzed. Additionally, high-throughput TCR- and BCR sequencing of the T- and B cell repertoire will be performed concerning the expansion of distinct clones and distribution of CDR3 sequences. To evaluate regulatory T-cell function levels of TGF-ß and IL-10 and the ability to suppress the activation and proliferation of effector T cells will be analyzed.

##### Proliferation assays

Human PBMCs will be labeled with 5 μM carboxyfluorescein succinimidyl ester (CFSE; Invitrogen) according to the manufacturer’s instructions. After stimulation with anti-human CD3 (2 μg/ml; OKT3) and soluble mouse anti-human CD28 (1 μg/ml; eBioscience) for 4 days proliferation will be assessed by flow cytometry. Otherwise, cell proliferation will be assessed using the ATPLite™ Luminescence Assay System (PerkinElmer) according to the manufacturer’s instructions. Luminescence will be measured on a TopCount NXT.

##### Cytokine detection

Appropriate ELISA-Kits will be used to detect cytokine concentrations in cell culture supernatants and serum. Alternatively, fluorescent bead immunoassays (FlowCytomix, eBioscience) will be used to quantify the production of the chemokines and cytokines according to the manufacturer’s instructions.

##### Intracellular calcium imaging

For intracellular calcium imaging experiments lymphocytes will be isolated using magnetic cell separation. All measurements will be performed in HEPES buffer containing (in mM): NaCl, 120; KCl, 2.5; NaH_2_PO_4_, 1.25; HEPES, 30; MgSO_4_, 2; glucose, 10; pH 7.25 and osmolarity will be set to 305 mOsm/kg. Cells will be loaded with 5 μM Fura-2 AM (Invitrogen) for 30 min at 37 °C. After 15 min anti-mouse CD3 (10 μg/ml; clone 145-2C11; ebioscience,) will be added and fluorescence will be measured using a TECAN infinite M200Pro fluorimeter (Tecan Group Ltd.). Excitation will be alternated between 340 and 380 nm and emission will be measured at 509 nm.

##### Migration assay

Transmigration assays will be performed with minor modifications as described previously [11]. In brief, HBMEC (human brain microvascular endothelial cells) will be cultured in transwell inserts until they reach confluency. Human PBMCs (each 5 × 10^5^) will be transferred to the HBMEC layer for 14 h. Migrated cells from the lower chamber will be collected and Calibrite beads (BD Biosciences) will be added. Next, human cells will be stained for CD4, CD8, CD14, CD19 and CD56, with the respective monoclonal antibodies and relative cell numbers were determined by flow cytometry.

##### High-throughput T-cell receptor sequencing

High-throughput sequencing (HTS) of the T-cell receptor (TCR) will be used to identify and quantify the distinct T-cell clone present within the biological samples. Therefore CDR3 region will be amplified and sequenced as described before [12, 13]. In brief, the method will use a multiplex PCR system to amplify all possible rearranges TCRβ CDR3 sequences from cDNA samples. Furthermore in a second step V and J gene primers will be used to amplify the rearranged VDJ segments which are subjected to HTS. Afterward the unique CD3 segment and the V,D, and J genes within each rearrangement will be identified and quantified using the ImmunoSEQ analyzer toolset and NCBI databases.

##### Suppression assay

CD4^+^CD25^+^CD127^dim/−^ regulatory T cells will be separated from responder T cells by magnetic-activated cell sorting using a CD4^+^CD25^+^CD127^dim/−^ Regulatory T Cell Isolation Kit II (Miltenyi Biotec). Responder T cells will be labeled with 5 μM carboxyfluorescein succinimidyl ester (CFSE; Invitrogen) according to the manufacturer’s instructions. Afterwards regulatory T cells and CFSE^+^ responder T cells will be cocultured at a ratio of 1:2 and 1:1, respectively. Cultures will be stimulated with anti-human CD3 (2 μg/ml; OKT3) and soluble mouse anti-human CD28 (1 μg/ml; eBioscience) for 4 days. After the stimulation period proliferation will be assessed by flow cytometry.

#### Additional endpoints

Furthermore, clinical related data as MRI scans and evaluation of disease activity and manifestation using EDSS and MSFC as well as data on number and time of relapses will be considered as additional endpoints.

##### Expanded disability status scale (EDSS)

Patient disability will be evaluated using the EDSS, which has long been considered the standard for assessing disability in patients with MS [14]. All involved physicians of the study team will be trained and certified to perform the EDSS in a consistent manner on www.neurostatus.net. The EDSS is an ordinal clinical rating scale which ranges from 0 (normal neurologic examination) to 10 (death due to MS) in half-point increments. Briefly, the assessing neurologist rates 7 functional systems (pyramidal, cerebellar, brainstem, sensory, bowel and bladder, visual, cerebral, and other) in the context of a standard neurological examination and then uses these ratings in conjunction with observations and information concerning the patient’s mobility, gait, and use of assistive devices to assign an EDSS score. EDSS steps 1.0 to 4.5 refer to people with MS who are fully ambulatory, while EDSS steps 5.0 to 9.5 are defined by the impairment to ambulation. The visual functional system score as determined as part of the assessments for EDSS will be considered as an additional endpoint.

##### Multiple sclerosis functional composite

The MSFC is a composite measure of impairment and acquisition of disability comprising quantitative tests of the function of the upper and lower limbs and cognitive abilities. Research to date has demonstrated that change in the MSFC over the first year of observation predicted a subsequent change in the EDSS, suggesting that the MSFC is more sensitive to change than the EDSS [14]. The Clinical Outcomes Assessment Task Force of the National MS Society developed the MSFC as a new scale for clinical outcomes measurements. The MSFC will be administered according to the MSFC manual of the National MS Society.

#### Sample size

At least 50 patients per group will be included in this study. The groups comprise three different groups: (i) de novo patients (until 04/2019), (ii) patients under alemtuzumab treatment and (iii) patients requiring more than two alemtuzumab infusions.

The sample size is justified by:

Cohen’s d (Δ/σ = difference of means/standard deviation) is a measure of effect size for mean differences of two groups with similar sample size. Cohen’s d is used to estimate the relevance of significant mean differences. The expected effect size can be estimated on the basis of data from previous studies:

1. Alemtuzumab leads to a decrease of absolute CD8^+^ T cell numbers in peripheral blood from 0,34 × 10^9^ ± 0,05 cells/L to 0,175 × 10^9^ ± 0,06 cells/L 6 months after treatment [15]. Here, Cohen‘s d equals 0,831.
2. Alemtuzumab increases the concentration of BDNF (brain-derived neurotrophic factor) produced by peripheral blood mononuclear cells under MBP (myelin basic protein) stimulation from 200 ± 82 pg/mL to 290 ± 73 pg/ml 6 months after treatment [15]. Here, Cohen’s d equals 0,502.

Clinical studies define effect sizes of Δ/σ = 0,2–0,3 as small, Δ/σ = 0,3–0,8 as medium and Δ/σ > 0,8 as large. Based on previous data we are expecting medium to large effect sizes with a Cohen’s d of > 0,5. With a type I error of α = 0,05 and a power of 0,95 the required sample size equals 42.

The precision of this estimation is considered to be sufficient to track the condition of the immune system over time to describe the mechanisms of action of alemtuzumab treatment.

#### Statistical methods

Statistical analyses will be performed using standard statistical software like SAS or SPSS. The goal of this study is to detect the patterns in different populations of immune cells after treatment with alemtuzumab. Therefore, the analysis of the primary endpoints will focus on estimating the absolute and relative number of cells defined as primary endpoints summary statistics such as mean and standard deviation, median and quartiles, or frequency and percent, as appropriate. The development over time will be displayed using boxplots. To assess the difference of absolute or relative number of cells between two different time points statistical tests appropriate to the statistical distribution of the particular endpoint will be used (Wilcoxon signed-rank test, Student’s t-test for paired samples, Sign-Test). In order to compare more than two successive measurements, the preferred methods of statistical analysis are Repeated Measures ANOVA, Mixed Models and Generalized Estimation Equations, appropriately accounting for intra-individual correlations. Since this study is planned as an exploratory study, inferential statistics are intended to be exploratory (hypotheses generating), not confirmatory, and are interpreted accordingly. I.e., *p*-values are interpreted as a metric weight of evidence against the respective null hypothesis of no effect. Neither a global significance level nor local levels are determined. *P*-values are considered noticeable in case p < =0.05 and highly noticeable in case p < =0.01. These findings will be used to generate new hypotheses. Statistical analyses of the pre-specified secondary endpoints will be performed with appropriate descriptive and inductive statistical methods using summary statistics such as mean and standard deviation, median and quartiles, or frequency and percent. Appropriate to the characteristics of the endpoints, statistical tests like Fisher’s exact test, Chi-Square test, Mann-Whitney-U test, Wilcoxon signed-rank test or Student’s t-test will be performed.

### Eligibility criteria

The below-mentioned eligibility criteria imply that they are indicated to receive or are already treated with alemtuzumab irrespective of study participation. Patients who do not meet all the inclusion criteria listed in Table 3 or meet one of the exclusion criteria listed in Table 3 will be excluded from further study participation.

**Table 3:** Eligibility criteria

Inclusion Criteria	
• Diagnosis of MS according to the McDonald criteria 2010 and cranial MRI scan demonstrating white matter lesions attributable to MS within 5 years before signing the informed consent form (ICF)	
• Age > 18 years	
• Written informed consent to study participation	
Exclusion Criteria	
• Medical, psychiatric, cognitive, or other conditions that, in the Investigator’s opinion, compromise the patient’s ability to understand the patient information, to give informed consent, or to complete the study	
• Any progressive form of MS	
• Any condition that serves as a contraindication for alemtuzumab treatment	
• Any disability acquired from trauma or another illness that could interfere with the evaluation of disability due to MS	
• Major systemic disease or other illness that would, in the opinion of the Investigator, compromise patient safety or interfere with the interpretation of study results, e.g., current peptic ulcer disease or other conditions that may predispose to hemorrhage	
• Significant autoimmune disease including but not limited to immune cytopenias, rheumatoid arthritis, systemic lupus erythematosus, other connective tissue disorders, vasculitis, inflammatory bowel disease, severe psoriasis	
• Inability to undergo MRI with gadolinium administration	

### Contacts

The study is an investigator initiated trial. Tobias Ruck, Heinz Wiendl and Sven Meuth developed the study design and protocol and are active clinical investigators in ProgramMS. The study will be performed in multiple KKNMS centres and is supported by the German Federal Ministry for Education and Research (BMBF).

## Perspective

Alemtuzumab is a highly effective disease modifying therapy for RRMS, which selectively targets CD52, an antigen presented on the surface of T- and B cells, the main drivers of pathology in MS. However, the exact mechanism of action remains largely unknown [9]. Our study aims to elucidate further insights into these mechanisms by performing a deep characterization of changes in immune cell distribution and function under alemtuzumab treatment in RRMS. Thereby, our study will help to develop new strategies for the prediction of potential side effects and to identify patients with optimal response improving the individual risk-benefit ratio for alemtuzumab therapy.

Alemtuzumab leads to a depletion of lymphocytes following a slow repopulation with a distinct sequential pattern: B cells reach baseline levels after 3 months, overshooting them at 6 months. In contrast T cells repopulate much slower and CD4^+^ and CD8^+^ T cells typically need several years to reach pre-treatment levels [9]. Besides quantitative changes of the immune cell repertoire, also qualitative alterations of immune components are observed, e.g. within the first month there is predominance of immature B cells, whereas the differentiation into memory B cells is very slow [15]. Therefore, it was proposed that alemtuzumab leads to a reconstitution of the immune system promoting tolerogenic mechanisms [9].

Interestingly, Jones et al. reported that specific changes in the reconstituting T-cell pool are associated with secondary autoimmunity. Patients with secondary autoimmunity had no greater T cell lymphopenia in comparison to those without but displayed higher T-cell apoptosis and cell cycling by genetically determined higher levels of IL-21. This enhanced cycling leads to an increased stochastic opportunity for T cells to encounter self-antigens thereby promoting autoimmunity [16]. Furthermore, T-cell recovery after alemtuzumab treatment is characterized by the proliferation of self-antigen responsive T cells, which have escaped the depletion. This so-called homeostatic proliferation leads to the generation of chronically activated (CD28^−^CD57^+^), highly proliferative (Ki67^+^), oligoclonal, memory-like CD4^+^ and CD8^+^ T-cells (CCR7^−^CD45RA^−^ or CCR7^−^CD45RA^+^) producing proinflammatory cytokines [17].

Those studies showed first insights into the changes in different immune-cell population. However, the exact changes in the immune cell profile including T- and B-cell populations at the same timepoints over a long period still need to be characterized.

Long-term remission and EDSS improvement under alemtuzumab therapy also fuelled the theory of additional neuroprotective effects beyond the well-known anti-inflammatory activity of alemtuzumab [18]. Jones et al. could show that peripheral blood mononuclear cells (PBMCs), isolated from MS patients treated with alemtuzumab, have increased levels of neurotrophic molecules leading to improved neuronal survival, axonal growth and oligodendrocyte survival and maturation in vitro [18]. However, this was only shown for peripheral immune cells. Moreover, an in vivo animal model of multiple sclerosis, the MOG_35–55_ EAE, failed to reproduce the neuroprotective effect driven by immune cell derived brain-derived neurotrophic factor (BDNF) [19]. The definite mechanism of action of the potential neuroprotective effects or whether there are such are still unknown and will be further investigated in this study by combining clinical data (EDSS, MSFC), MRI (brain atrophy, structural integrity) and immunological analysis (ex vivo and in vitro data).

Recently, a safety review of the European Medicines Agency’s (EMA) was initiated since 39 patients had suffered from vascular events such as heart attacks or strokes under therapy with alemtuzumab. Currently, the review is still ongoing and the final report is anticipated by the end of the year [8]. However, secondary autoimmunity seems to remain the most relevant adverse effect of alemtuzumab with thyroid disorders, immune thrombocytopenia and immune-mediated nephropathies being most prevalent with the highest incidence 1–3 years after treatment initiation [20]. Biomarkers allowing to identify patients at risk for the development of secondary autoimmune diseases are currently not available. Therefore, new biomarkers and/or immune-signatures predicting secondary autoimmunity are needed to improve the safety profile of alemtuzumab. Our study will use in depth analysis of immune cell status over time to reveal potential new signatures of immune reconstitution, that might be able to predict secondary autoimmunity. However, since the study is limited to three years follow up, further validation of potential biomarkers will be needed for the long-term treatment with alemtuzumab.

### Limitations of the study

The approach of a multicentre study results in a higher rate of patient enrolment and improves the generalizability of the study. Nevertheless, multicentre studies have several disadvantages regarding the difference in the severity of the disease and the heterogeneity in clinical practice among centers, which is a major confounding factor for the interpretation of the results [21]. Therefore, a rigorous study protocol for uniform data collection was created. However, patient recruitment takes place in highly specialized centers for multiple sclerosis (centers of the Kompetenznetz Multiple Sklerose KKNMS) which might bias the patient pool, because these patients might have a more complicated and severe disease history, which could limit our results at least in part.

Furthermore, the outcome of the study might be influenced by the potential loss of follow ups. Patients who discontinue study participation or the treatment with alemtuzumab prematurely will not be replaced. To avoid an effect on the results we calculated that a sample size with at least 50 patients per each group (de novo patients; patients under alemtuzumab treatment; patients requiring more than two alemtuzumab infusions) is necessary to compensate potential loss of follow up.

Although we aim to elucidate the mechanism of action of the potential immune-reconstitutive effects of alemtuzumab within this study, the results might be limited since the data will only be generated from blood samples. Since the pathologic process of MS takes place in the CNS, it is still unknown how representative the lymphocyte populations/parameters in the peripheral blood samples may be, since the transmigration via the blood-brain barrier is a tightly regulated process and might be affected in disease and under therapy [22]. Further studies will be needed to characterize the immunological change and explore new biomarkers in the brain itself using animal models or potentially CSF analysis. However, the requirement for lumbar puncture constitutes a major barrier for more general use, especially when repeated lumbar punctures are needed.

## Summary

Alemtuzumab is a highly effective therapy in relapsing-remitting multiple sclerosis (RRMS). However, the exact mechanisms of action remain to be understood. Therefore, this study aims to further elucidate these mechanisms in RRMS treatment and to develop new strategies for the prediction of the risk-benefit ratio for individual patients. The currently ongoing safety review by European authorities further supports the urgent need for such strategies. For the study blood samples of RRMS patients treated with alemtuzumab will be analyzed semi-annually up to 36 months. Using in vitro and ex vivo assays we aim to deeply characterize immune cell populations including their functional activity. Combining these analyses with clinical data (MRI, EDSS, MSFC) will help to reveal the underlining mechanism related to efficacy and safety of alemtuzumab.

## Data Availability

Not applicable.

## Abbreviations

BMBF: German Federal Ministry for Education and Research
CNS: Central Nervous System
EDSS: Expanded Disability Status Scale
EMA: European Medicine Agency
HBMEC: Human Brain Microvascular Endothelial Cells
KKNMS: Kompetenznetzwerk Multiple Sklerose
MRI: Magnetic Resonance Imaging
MSFC: Multiple Sclerosis Functional Composite
PBMC: Peripheral Blood Mononuclear Cells
RRMS: Relapsing-Remitting Multiple Sclerosis
SAD: Sustained Accumulation of Disability
SPMS: Secondary progressive Multiple Sclerosis
